# A Rhodamine B-Based “Turn-On” Fluorescent Probe for Selective Fe^3+^ Ions Detection

**DOI:** 10.3390/s25113477

**Published:** 2025-05-31

**Authors:** Md Foridul Islam, Abdulkadir Zakari Abdulkadir, Smaher M. Elbayomi, Pengfei Zhang

**Affiliations:** 1 Guangdong Key Laboratory of Nanomedicine, CAS-HK Joint Laboratory of Biomaterials, CAS Key Laboratory of Biomedical Imaging Science and System, Institute of Biomedicine and Biotechnology, Shenzhen Institutes of Advanced Technology (SIAT), Chinese Academy of Sciences, Shenzhen 518055, China; humaunahammedforid@gmail.com (M.F.I.); abdulkadirzakariabdulkadir@gmail.com (A.Z.A.); 2 University of Chinese Academy of Sciences, Beijing 101408, China; 3 Polymer Institute, Slovak Academy of Sciences, Dúbravská Cesta 9, 845 41 Bratislava, Slovakia; 4 Department of Chemistry, Faculty of Science, Damietta University, New Damietta 34517, Damietta, Egypt

**Keywords:** rhodamine B, fluorescent probe, turn-on fluorescence, metal ions detection, reversible fluorescent, environmental monitoring

## Abstract

Detecting heavy metal ions is essential for maintaining environmental safety, ensuring industrial quality control, and protecting public health. In this study, we have synthesized a novel Rhodamine B-based fluorescent probe, RhB-DCT, which is functionalized with 2,4-dichloro-1,3,5-triazine (DCT) to enhance selectivity and sensitivity for metal ions detection. The probe functions through a “turn-on” fluorescence mechanism activated by the opening of the spiro-lactam ring induced by Fe^3+^ ions, resulting in a distinct color change from colorless to deep pink. The RhB-DCT probe demonstrated a rapid and robust fluorescence response within seconds, exhibited a broad pH stability from 4 to 13, showed excellent reversibility, and possessed a low detection limit of 0.0521 μM, surpassing numerous existing fluorescent probes. The RhB-DCT probe exhibited significant selectivity for Fe^3+^ than other competing metal ions. The integration of high sensitivity, rapid response, and strong stability positions RhB-DCT as a viable option for real-time detection of Fe^3+^ ions in aqueous settings. This study demonstrates the efficacy of the RhB-DCT probe in environmental monitoring, water quality assessment, and analytical sensing platforms, serving as an effective and dependable tool for detecting heavy metal ions.

## 1. Introduction

Heavy metal ions contamination of water sources presents a significant risk to ecosystems and human health, attributed to their high toxicity, persistence, and non-biodegradability [1,2,3]. Heavy metals including Lead (Pb^2+^), Mercury (Hg^2+^), Cadmium (Cd^2+^), Chromium (Cr^3+^), Copper (Cu^2+^), Zinc (Zn^2+^), and Iron (Fe^3+^) are commonly detected in industrial effluents, agricultural runoff, and natural water bodies [4,5,6,7,8,9,10]. Trace levels of these ions can lead to significant environmental harm and long-term health issues, such as neurotoxicity, renal failure, and cancer [11,12]. Developing sensitive, selective, and rapid methods for detecting heavy metal ions is crucial for environmental monitoring, public safety, and biomedical research.

Iron ions (Fe^3+^) hold a distinct role among these metals. Fe^3+^ is essential in biological systems for functions such as oxygen transport, enzymatic reactions, and DNA synthesis [13]. However, its excessive accumulation can lead to oxidative stress, organ dysfunction, and neurodegenerative diseases, including Alzheimer’s and Parkinson’s [14]. Excessive levels of Fe^3+^ in environmental water can disrupt aquatic ecosystems and contaminate drinking water supplies, highlighting the importance of sensitive detection of Fe^3+^ [15].

Traditional detection techniques for metal ions detection, such as atomic absorption spectroscopy (AAS), inductively coupled plasma mass spectrometry (ICP-MS), and electrochemical analysis, etc., provide high accuracy and low detection limits [16,17,18]. These techniques always need costly instrumentation, complex sample preparation, and skilled personnel, thereby constraining their practical use for routine or field monitoring. Consequently, there is an increasing demand for efficient, economical, and real-time sensing methodologies.

Fluorescent chemo-sensors have garnered considerable interest as effective tools for the detection of heavy metal ions, attributed to their simplicity, rapid response, high sensitivity, and capability for real-time monitoring [19,20,21]. Rhodamine-based fluorescent probes are significant due to their high photostability, excellent fluorescence quantum yield, and a unique “off–on” fluorescence switching mechanism that relies on a metal ion-induced spiro-lactam ring opening [22,23,24].

In fluorescent sensing, “turn-on” systems present distinct advantages compared to “turn-off” mechanisms [25]. Turn-off probes frequently experience background fluorescence quenching due to environmental factors, resulting in false negatives or diminished signal-to-noise ratios [26,27]. In contrast, turn-on probes produce a bright, detectable signal exclusively upon binding with the target ions, thereby reducing false readings and enhancing detection reliability, especially at trace concentrations [28,29,30].

**Figure 1:**
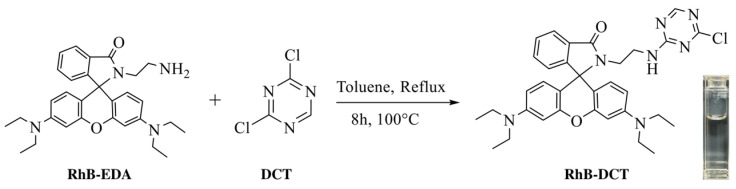
Synthetic routes of the RhB-DCT fluorescent probe.

Despite notable advancements, numerous rhodamine-based probes exhibit limitations such as restricted pH stability, prolonged response times, inadequate reversibility, and insufficient selectivity, particularly in intricate aqueous settings. In response to these challenges, we designed and synthesized a novel Rhodamine B-based fluorescent probe, functionalized with 2,4-dichloro-1,3,5-triazine (DCT), termed RhB-DCT (See Figure 1). The triazine group, recognized for its strong electron-withdrawing characteristics and reactivity, was incorporated to improve the binding affinity, selectivity, and chemical stability of the probe, especially in aqueous conditions. The structural modification facilitates a spiro-lactam ring-opening mechanism, leading to a notable “turn-on” fluorescence and colorimetric response upon interaction with Fe^3+^ ions. The evaluation of the RhB-DCT probe identified several notable characteristics: a rapid and significant fluorescence enhancement upon binding with Fe^3+^, swift reaction kinetics achieving equilibrium within seconds, high stability over a broad pH range, good reversibility during chelation and dechelation cycles, and a remarkably low detection limit. The probe exhibited significant selectivity for Fe^3+^, even amidst competing metal ions. The results demonstrate the efficacy of the RhB-DCT probe as a sensitive and reliable instrument for real-time detection of Fe^3+^ in water samples, contributing to environmental monitoring and analytical sensing applications.

## 2. Materials and Methods

### 2.1. Materials

Rhodamine B (RhB) was purchased from Shanghai Bidepharm Co., Ltd. (Shanghai, China); Ethylenediamine (EDA) was purchased from Acros Organics (Waltham, MA, USA); 2,4-Dichloro-1,3,5-triazine (DCT) was purchased from Shanghai Macklin Biochemical Co., Ltd. (Shanghai, China); Hydrochloric Acid (HCl), Ethanol Absolute (EtOH), Dichloromethane (DCM), Toluene and other reagents were used without further purification. Metal ions such as Ca^2+^, Mg^2+^, K^+^, Hg^2+^, Fe^3+^, Co^2+^, Mn^2+^, Cd^2+^, Al^3+^, Ni^2+^, Na^+^, Cu^2+^, Ag^+^, Pb^2+^, Li^+^, and Zn^2+^ were prepared from their Cl^−^ and SO_4_^2−^ salts in the laboratory. Various common anions such as AcO^−^, B_4_O_7_^2−^, Br^−^, CO_3_^2−^, Cl^−^, F^−^, H_2_PO_4_^−^, HCO_3_^−^, I^−^, NO_3_^−^ PO_4_^3−^, S^2−^, and SO_4_^2−^ were also prepared from their corresponding Na and K salts in the laboratory.

### 2.2. Synthesis of RhB-DCT Fluorescent Probe

First, RhB-EDA conjugate was synthesized following a modified version of the procedure [31]. Then, 1.44 g of RhB-EDA and 0.44 g of 2,4-dichloro-1,3,5-triazine (DCT) were precisely measured and dissolved in 50 mL of toluene. The solution was refluxed in a condensation setup with continuous stirring at 300 RPM and a temperature of 100 °C for 8 h to promote the reaction. After completion, the mixture was permitted to cool to room temperature naturally. The solvent was subsequently eliminated under reduced pressure through rotary evaporation. Then the crude residue was dissolved in DCM and subsequently purified through column chromatography on a silica gel column, employing a 1:1 mixture of ethyl acetate and petroleum ether as the mobile phase. The product, RhB-DCT, was obtained as a white solid with a yield of 0.66 g. The reaction’s success was verified using ^1^H NMR spectroscopy (Figure S1), ^13^C NMR spectroscopy (Figure S2), and Fourier-transform infrared (FTIR) spectroscopy (Figure S3). ^1^H NMR (400 MHz, CDCl_3_) δ 8.23, 8.23, 7.93, 7.92, 7.91, 7.91, 7.90, 7.48, 7.47, 7.47, 7.46, 7.46, 7.45, 7.45, 7.11, 7.10, 7.09, 7.08, 7.08, 7.05, 7.03, 6.41, 6.41, 6.39, 6.38, 6.37, 6.36, 6.36, 6.35, 6.23, 6.23, 6.21, 6.20, 3.43, 3.42, 3.41, 3.40, 3.40, 3.38, 3.35, 3.34, 3.32, 3.32, 3.30, 3.26, 3.25, 3.23, 3.22, 3.20, 3.19, 3.18, 3.16, 1.18, 1.17, 1.16, 1.16, 1.15, 1.14. ^13^C NMR (101 MHz, CDCl_3_) δ 170.40, 169.54, 166.89, 165.20, 153.71, 153.39, 148.98, 132.92, 130.62, 128.52, 128.30, 123.97, 123.06, 108.22, 104.87, 97.75, 65.60, 44.43, 41.73, 39.09, 12.67.

### 2.3. Characterization and Instruments

^1^H-NMR and ^13^C-NMR spectra were acquired on a Bruker 400 MHz spectrophotometer (Bruker BioSpin AG, Fällanden, Switzerland) in CDCl_3_. Absorption spectra were measured on a Supermax 2800MF spectrophotometer (Shimadzu Corporation, Kyoto, Japan). Fluorescence spectra measurements were performed on a Techcomp FL970 spectrofluorometer (Shanghai Haitian Scientific Instrument Co., Ltd., Shanghai, China). The FTIR Spectrum was measured by a Nicolet iN10 Infrared Microscope (Thermo Fisher Scientific, Waltham, MA, USA). Macroscopic pictures were taken under 365 nm UV light to investigate the fluorescence characteristics of the samples.

## 3. Results and Discussion

### 3.1. Detection of Metal Ions

To evaluate the sensing performance of the newly synthesized RhB-DCT probe, a stock solution was prepared at a concentration of 1 × 10^−3^ M in absolute ethanol, which was subsequently diluted to 5 × 10^−5^ M for further optical analysis. Metal ion solutions at a concentration of 1 × 10^−3^ M were prepared using double-distilled water and then diluted to 1 × 10^−4^ M. These solutions were subsequently added to the RhB-DCT solution in an ethanol–water mixture (1:1, *v*/*v*) adjusted to a neutral pH of 7.0. The RhB-DCT probe solution was colorless under ambient conditions, suggesting the absence of structural transformation or metal-induced interaction within the solvent system alone.

**Figure 2:**
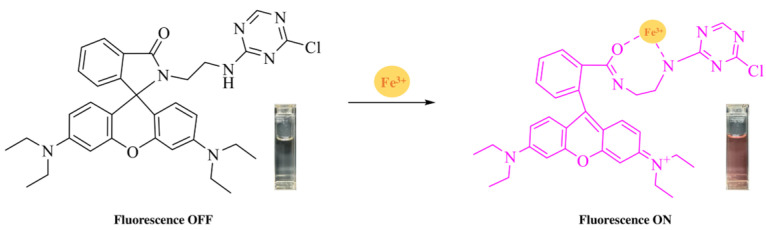
The proposed binding mechanism of RhB-DCT and Fe^3+^.

Upon the addition of Fe^3+^ ions and a subsequent 10 min incubation, a noticeable color change from colorless to deep pink was observed, indicating a significant interaction between Fe^3+^ and the RhB-DCT molecule. The observed color shift is due to the opening of the spiro-lactam ring structure of RhB-DCT, which generally remains closed and non-fluorescent until it coordinates with specific metal ions, as shown in Figure 2. Al^3+^ ions induced a minor alteration in hue, while the introduction of other metal ions under identical experimental conditions resulted in no significant visual change.

The initial observations highlight the selectivity of the RhB-DCT fluorescent probe for Fe^3+^ ions, exhibiting minimal interference from other assessed metal species. The pronounced color change observed with the addition of Fe^3+^ indicates the potential application of RhB-DCT as an effective and selective colorimetric sensor for Fe^3+^ in aqueous and semi-aqueous environments.

**Figure 3:**
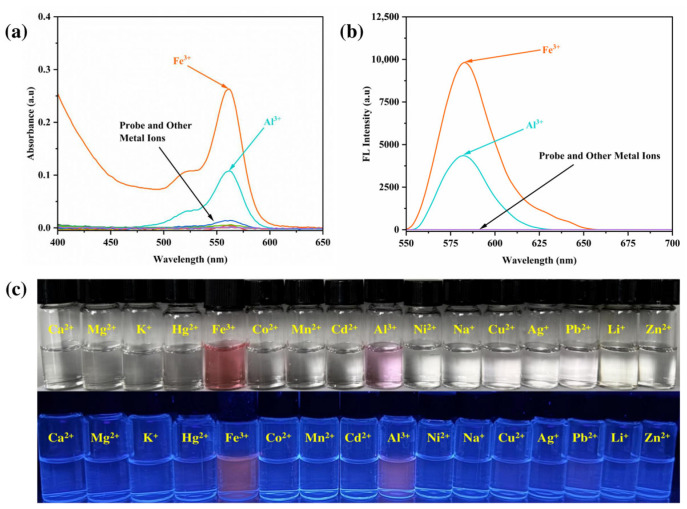
(**a**) UV-vis absorption spectra and (**b**) ffluorescence spectra of RhB-DCT probe upon the addition of various metal ions. (**c**) Macroscopic pictures under sunlight (up) and ultraviolet light (down) of RhB-DCT probe for detection of different metal ions.

UV-vis absorption spectroscopy was employed to further investigate the metal ion recognition behavior of the RhB-DCT probe. The spectral response exhibited a significant absorption band that increased at approximately 562 nm, observed solely with the addition of Fe^3+^ ions, as shown in Figure 3a. The emergence of a strong absorbance signal indicates that RhB-DCT demonstrates significant sensitivity and selectivity for Fe^3+^ ions. The introduction of Al^3+^ ions resulted in a marginal increase in absorbance intensity, while the other metal ions tested produced minimal to no spectral variation within the same wavelength range. The findings indicate that RhB-DCT experiences a specific structural or electronic transition upon interaction with Fe^3+^, while remaining unresponsive to other competing metal ions. The significant response at 562 nm confirms the probe’s selective recognition capability under neutral aqueous-organic conditions.

The interaction of the RhB-DCT probe with Fe^3+^ ions was validated through fluorescence spectroscopy at an excitation wavelength of 550 nm. An intense emission peak at 584 nm was observed upon the addition of Fe^3+^ ions, as shown in Figure 3b. The significant rise in fluorescence intensity indicates the activation of the probe’s emissive properties upon complexation with Fe^3+^, implying a transformation in its molecular structure, likely resulting from the opening of the spiro-lactam ring. The addition of Al^3+^ also resulted in a slight increase in fluorescence intensity, whereas other metal ions showed no prominent results, demonstrating the probe’s selective responsiveness to Fe^3+^ in the ethanol–water system.

Visual evaluation of the RhB-DCT-based probe system was conducted under both sunlight and ultraviolet (UV) light to assess its response to different metal cations, as shown in Figure 3c. The probe solution initially remained colorless in natural light, suggesting that the rhodamine framework predominantly existed in its non-fluorescent, closed spiro-lactam form. Upon exposure to Fe^3+^ ions, the solution underwent an immediate and vivid color change to deep pink, clearly noticeable by the naked eye, implying the formation of a colored complex due to ring opening and strong interaction with the metal ion.

Under UV light (365 nm), the fluorescence behavior of the system became more prominent and metal ion-dependent. A substantial fluorescence enhancement was observed only in the presence of Fe^3+^, which also contributed to the intensification of the pink color. This visual fluorescence provided additional confirmation of Fe^3+^-induced structural transition within the probe. Meanwhile, the addition of Al^3+^ led to a slight increase in fluorescence, with minimal impact on the overall emission profile. Other metal ions triggered negligible optical or fluorescent shifts, indicating the probe’s selectivity toward Fe^3+^ ions.

These distinct chromatic and photoluminescent responses demonstrated the RhB-DCT system’s potential as a colorimetric and fluorometric indicator for Fe^3+^ detection. Its ability to exhibit visible changes without sophisticated instrumentation makes it particularly promising for field-based analysis, real-time monitoring, and visual detection in environmental applications. The clear color differentiation and selective fluorescence enhancement offer a user-friendly and practical approach for the qualitative identification of Fe^3+^ ions, with potential extensions into areas such as pollutant screening and environmental monitoring.

### 3.2. Analysis of Sensing Characteristics

Competitive ion experiments were conducted to enhance understanding of the selectivity and interference resistance of the RhB-DCT probe system. The fluorescence response of the probe was evaluated under two conditions: in the presence of individual metal ions and in mixtures where each ion was co-introduced with Fe^3+^ at equal concentrations.

**Figure 4:**
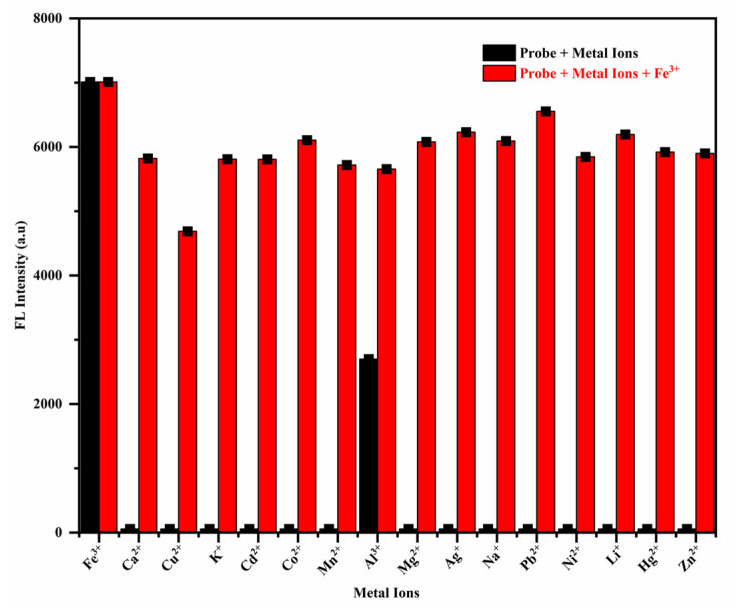
Fluorescence intensity for comparative investigation between Fe^3+^ and other metal ions.

The experimental setup was kept consistent to ensure comparability in this analysis. As shown in Figure 4, the black bars represent the fluorescence intensity produced by the probe when exposed to each metal ion individually, while the red bars indicate the fluorescence intensity upon the addition of Fe^3+^ alongside these ions. The results indicated that the majority of co-existing metal ions exerted minimal influence on the fluorescence emission triggered by Fe^3+^, thereby confirming the RhB-DCT probe’s high selectivity for Fe^3+^, even amidst potentially competing ions.

As shown in Figure S4, the visual response of the RhB-DCT solution under sunlight (up) and UV light (down) highlights the distinct optical changes induced by different metal ions. The introduction of Fe^3+^ ions resulted in a distinct pink coloration, markedly different from the subtle color changes noted with other metal ions. This contrast highlights the strong affinity of the RhB-DCT probe for Fe^3+^, enabling a distinct and selective binding interaction.

Subsequent spectroscopic analysis corroborated these visual observations. The presence of Fe^3+^ markedly increased the fluorescence intensity relative to the blank solution. Measurements at 550 nm indicated that the probe’s interaction with Fe^3+^ produced a significantly stronger signal compared to other coexisting metal ions. While other ions induced minor alterations, their impacts were significantly less pronounced and did not disrupt the Fe^3+^-induced response. In mixed-ion conditions, the color and fluorescence output closely resembled that of the Fe^3+^-only solution, though with slightly reduced intensity, thereby confirming the probe’s selectivity and sensitivity. The results indicate that the RhB-DCT probe is an effective and visually intuitive tool for detecting Fe^3+^ ions, even in complex environments with various competing metal species.

### 3.3. Effect of Common Anions on the Fluorescent Properties of RhB-DCT-Fe^3+^ Complex

**Figure 5:**
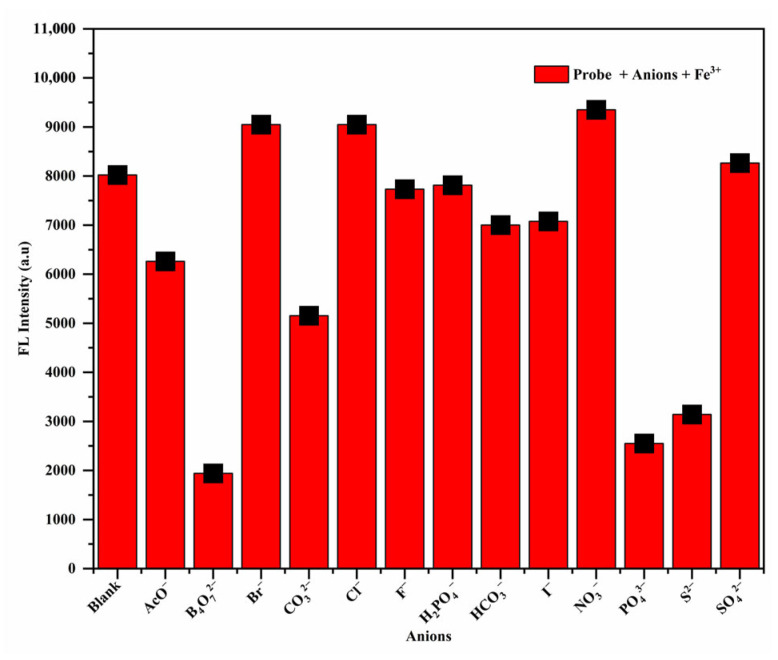
Fluorescence changes of the solution containing RhB-DCT and Fe^3+^ upon the addition of various common anions.

Furthermore, fluorescence spectroscopy was employed to evaluate the impact of different anions on the RhB-DCT-Fe^3+^ complex. A diverse array of anions was evaluated, including AcO^−^, B_4_O_7_^2−^, Br^−^, CO_3_^2−^, Cl^−^, F^−^, H_2_PO_4_^−^, HCO_3_^−^, I^−^, NO_3_^−^, PO_4_^3−^, S^2−^, and SO_4_^2−^. As shown in Figure 5, the fluorescence spectra demonstrated differential effects based on the particular anion involved. For example, B_4_O_7_^2−^, PO_4_^3−^, and S^2−^ resulted in a notable decrease in fluorescence intensity, indicating possible interference with the binding of Fe^3+^ to the RhB-DCT probe. Conversely, Br^−^, Cl^−^, and NO_3_^−^ led to a significant enhancement in fluorescence emission, possibly due to their effect on the local microenvironment (e.g., polarity) that promotes the fluorescent ring-opened state demonstrating a beneficial effect on the probe’s fluorescence characteristics. The remaining anions, including AcO^−^, CO_3_^2−^, F^−^, H_2_PO_4_^−^, SO_4_^2−^, HCO_3_^−^, and I^−^ did not produce significant alterations in fluorescence intensity under identical experimental conditions. The observations highlight the selective interaction of the RhB-DCT probe with Fe^3+^, despite the presence of competing anions, thereby underscoring its potential for specific metal ions detection.

### 3.4. Binding Stoichiometry and the Association Constant

**Figure 6:**
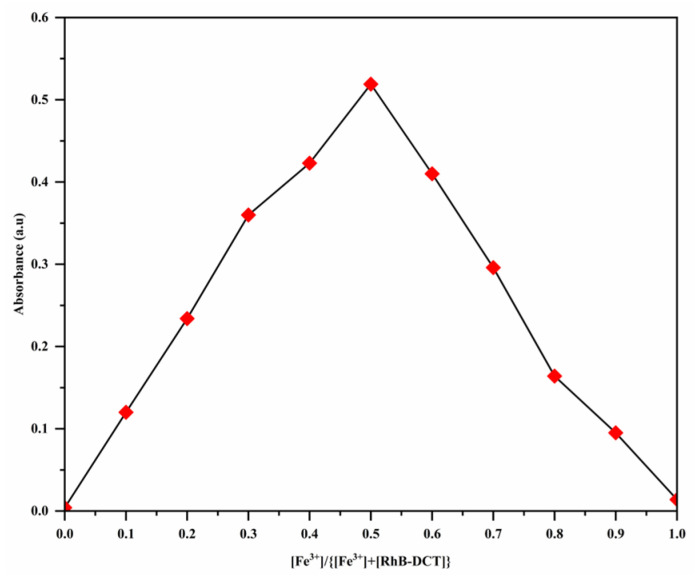
Job’s plot for the stoichiometry of RhB-DCT and Fe^3+^ at 562 nm in an ethanol–water solution (*v*:*v* = 5:5).

The binding interaction between Fe^3+^ ions and the RhB-DCT probe was investigated using the equimolar continuous variation technique, known as Job’s method. This method determines the stoichiometric ratio of complex formation. The total molar concentration of RhB-DCT and Fe^3+^ was maintained at 50 μM, with the mole fraction of Fe^3+^ systematically varied from 0.0 to 1.0. All solutions were prepared using a 1:1 ethanol–water mixture to ensure consistent solvent conditions. UV-vis spectrophotometry was employed to observe changes in absorbance, specifically targeting the absorption band at 562 nm, which signifies the complexation of RhB-DCT-Fe^3+^. As shown in Figure 6, the Job’s plot, which was created by plotting absorbance values against the mole fraction of Fe^3+^, shows a maximum intensity at a mole fraction of 0.5. The peak indicates a 1:1 molar interaction between RhB-DCT and Fe^3+^, resulting in a stable coordination complex. The findings indicate a selective and defined binding mechanism between the RhB-DCT probe and Fe^3+^ ions.

The binding stoichiometry of RhB-DCT and Fe^3+^ ions has been verified using the Benesi–Hildebrand method, which suggests a 1:1 host–guest complex. The following Benesi–Hildebrand equation was applied:

$$\frac{1}{I-I_{0}}=\frac{1}{K_{a}(I_{\max}-I_{0})[{Fe}^{3+}]}+\frac{1}{I_{\max}-I_{0}}$$

where the absorbance intensities of the RhB-DCT solution in the presence and absence of Fe^3+^ are indicated as I and I_0_, respectively. I_max_ is the absorbance at saturation with excess Fe^3+^, Ka is the association constant, and [Fe^3+^] is the concentration of Fe^3+^ ions in μM [32]. Figure S5 demonstrates the plot’s linearity (R^2^ = 0.9961), which strongly supports a 1:1 binding stoichiometry. The plot’s slope-to-intercept ratio showed a binding constant (K_a_) of 3.22 × 10^4^ M^−1^, indicating a significant interaction between the probe and Fe^3+^ ions.

### 3.5. Impact of Different Concentrations

**Figure 7:**
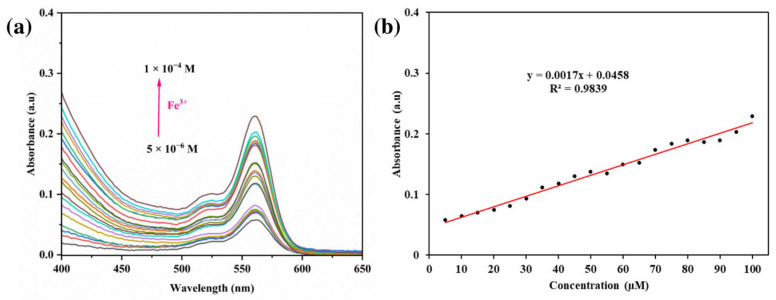
(**a**) UV-vis absorption spectra of RhB-DCT probe upon the addition of Fe^3+^ with various cconcentrations. (**b**) Changes of fluorescence absorbance as a function of Fe^3+^ concentration.

The impact of different Fe^3+^ ions concentrations on the optical response of the RhB-DCT fluorescent probe was systematically investigated, as shown in Figure 7a. The probe exhibited significant sensitivity to Fe^3+^, especially within the UV-visible absorption spectrum. The gradual addition of Fe^3+^ within the concentration range of 5 μM to 100 μM (5.0 × 10^−6^ M to 1.0 × 10^−4^ M) resulted in a significant and progressive increase in absorbance at 562 nm. The observed spectral shift correlated directly with elevated Fe^3+^ concentrations, signifying a robust and specific interaction between the metal ions and the RhB-DCT probe. A clear linear relationship (R^2^ = 0.9839) was observed between absorbance intensity and Fe^3+^ concentration, as shown in Figure 7b. The linearity highlights the RhB-DCT system’s ability to quantitatively detect Fe^3+^ ions across a broad concentration range, indicating its potential as a reliable and efficient platform for Fe^3+^ ions detection, with enhanced sensitivity relative to previously reported systems.

**Figure 8:**
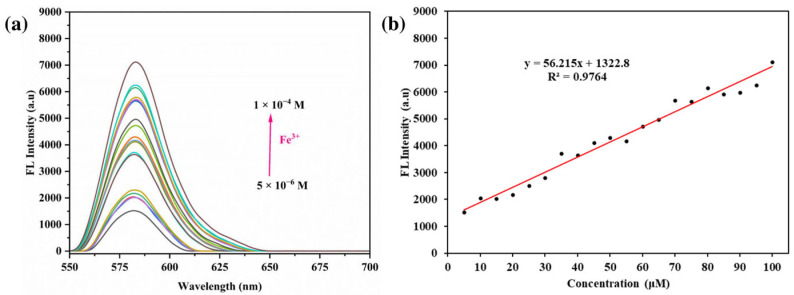
(**a**) Fluorescence spectra of RhB-DCT probe upon the addition of Fe^3+^ with various cconcentrations. (**b**) Changes of fluorescence intensity as a function of Fe^3+^ concentration.

After that, emission spectra were recorded to examine the fluorescence response of the RhB-DCT system to varying concentrations of Fe^3+^ ions, specifically within the range of 10 to 100 μM. As shown in Figure 8a, the fluorescence intensity increased consistently with higher Fe^3+^ concentrations, indicating a robust interaction between the RhB-DCT probe and the target ions. The emission maximum was consistently observed at 584 nm, and a direct linear correlation was established within the studied range, resulting in a correlation (R^2^ = 0.9764), as shown in Figure 8b. The probe’s sensitivity was quantified by calculating the Limit of Detection (LOD), which was found to be approximately 0.0521 μM. The LOD was calculated based on the relationship defined by LOD = 3 × δ_blank_/K (where, δ_blank_ is the standard deviation of the blank solution and K is the slope of the calibration curve) [33]. This low detection threshold highlights the enhanced analytical performance of the RhB-DCT probe for detecting Fe^3+^ in comparison to numerous previously reported systems, as shown in Table 1.

Furthermore, visual assessment corroborated the spectroscopic data, indicating that both UV absorption and fluorescence output intensified by increasing Fe^3+^ concentrations. Macroscopic color changes and gel-phase imaging under sunlight further confirmed the findings, consistent with the spectral measurements. The results demonstrate the high sensitivity and selectivity of the RhB-DCT system, along with its reproducibility and practical applicability for the visual and optical detection of Fe^3+^ ions in solution.

### 3.6. Impact of Reaction Time

**Figure 9:**
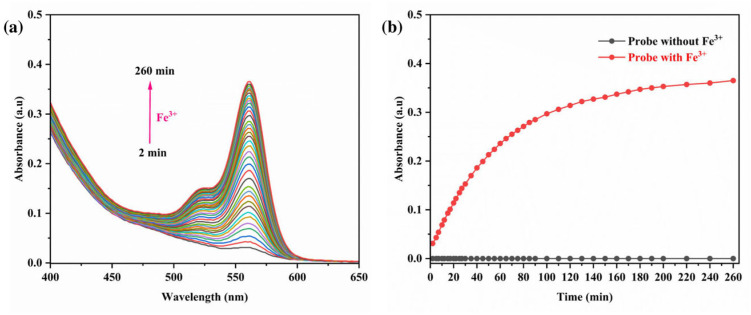
(**a**) UV-vis absorption spectra of RhB-DCT probe upon the addition of Fe^3+^ with various reaction times. (**b**) Impact of reaction times on the fluorescence absorbance of the RhB-DCT probe in the presence and absence of Fe^3+^.

A time-dependent UV-vis spectroscopic study was conducted to assess the impact of chelation duration on the efficacy of the RhB-DCT fluorescent probe in detecting Fe^3+^ ions. A mixture of equal volumes of RhB-DCT solution (5 × 10^−5^ M) and FeCl_3_ solution (1 × 10^−4^ M) was prepared, and spectral changes were monitored at regular intervals. As shown in Figure 9a, there was a progressive increase in UV absorbance at the characteristic wavelength over time, alongside a noticeable transition in color to pink. The absorption reached a plateau after about 100 min, signifying the completion of the coordination interaction between Fe^3+^ ions and the RhB-DCT probe, as shown in Figure 9b. The time-dependent behavior confirmed that the chelation process progresses consistently until equilibrium is achieved, underscoring the probe’s gradual and stable complex formation in solution.

**Figure 10:**
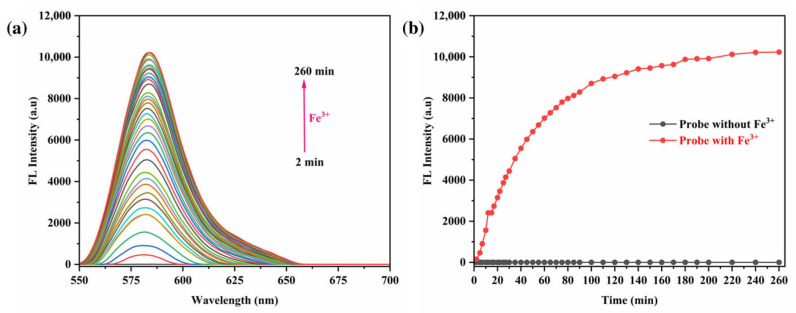
(**a**) Fluorescence spectra of RhB-DCT probe upon the addition of Fe^3+^ with various reaction times. (**b**) Impact of reaction times on the fluorescence intensity of the RhB-DCT probe in the presence and absence of Fe^3+^.

Furthermore, fluorescence emission studies were performed to observe the temporal dynamics of the interaction between the RhB-DCT probe and Fe^3+^ ions. As shown in Figure 10a, the fluorescence intensity increased steadily and consistently with the prolongation of the chelation period. At an excitation wavelength of 550 nm, the emission intensity increased consistently, reaching a saturation point approximately at 100 min, indicating the completion of complex formation, as shown in Figure 10b. The observed enhancement in fluorescence suggests that the temporal evolution of the system is dictated by the binding kinetics of RhB-DCT with Fe^3+^. The results indicate that extended contact time enhances interaction, thereby affecting the photophysical properties and overall efficacy of the probe in detecting Fe^3+^ ions.

### 3.7. The pH Effect

**Figure 11:**
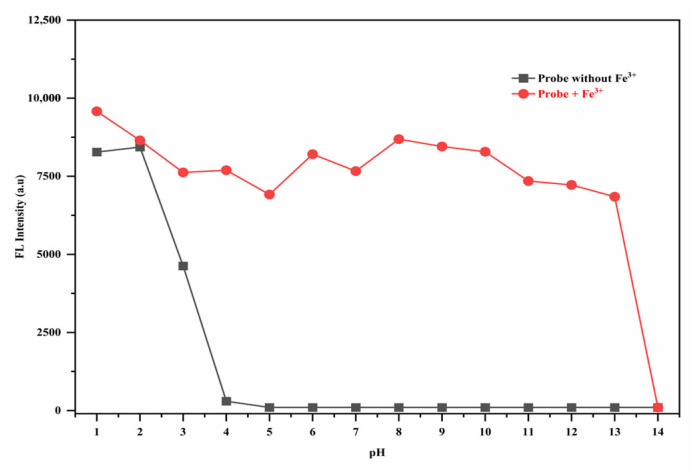
Effects of pH on the fluorescence spectra of RhB-DCT and RhB-DCT + Fe^3+^ in a 5:5 (*v*:*v*) ethanol–water solution.

The RhB-DCT fluorescent probe demonstrated distinct pH-dependent photophysical characteristics, demonstrating its capability for sensitive monitoring of acidic environments. First of all, the pH levels were adjusted with commercially prepared buffer solutions (pH 1–14). We used 200 µL of buffer solution in each sample for accurate and consistent pH control. The final pH values were verified with a calibrated digital pH meter. As shown in Figure 11, the fluorescence emission increased progressively as the environmental pH decreased from 4.0 to 1.0, independent of Fe^3+^ ion presence. Here, the spiro-lactam moiety’s ring probably opened as a result of protonation. Above a pH of 4.0, the probe’s fluorescence was predominantly quenched, indicating limited spectral activity. The enhancement observed at lower pH values is due to the acid-induced opening of the spiro-lactam ring structure of RhB-DCT, which is initiated by protonation in strongly acidic conditions. The probe’s sensitivity to minor pH variations, especially within the low-pH spectrum, highlights its adaptability and appropriateness for use in proton-rich or biologically acidic settings.

The interaction of Fe^3+^ ions with the RhB-DCT fluorescent system across a pH range of 1.0 to 13.0 demonstrated significant alterations in fluorescence emission and visible color response. A significant increase in fluorescence intensity was observed between pH 4.0 and 13.0, demonstrating that RhB-DCT maintained its effective sensing capabilities across mildly acidic to basic conditions. The activation responsive to pH is due to the Fe^3+^-induced opening of the probe’s spirocyclic structure, which promotes intramolecular charge transfer and emission. At pH levels above 13.0, a significant reduction in fluorescence output was noted to pH 14.0, presumably due to the precipitation of Fe(OH)_3_, which disrupts the probe–ion interaction, as shown in Figure S6. The results indicate that RhB-DCT serves as an effective pH-tolerant chemo-sensor for Fe^3+^ ions.

The RhB-DCT system’s capacity to adjust its emission characteristics under different pH conditions demonstrates its adaptability for use in complex environments. The consistent optical response across physiological and environmental pH ranges renders it appropriate for metal ion detection and as a pH-sensitive fluorescent indicator in biochemical assays, environmental diagnostics, and various pH-dependent sensing applications.

### 3.8. The Effect of the Temperature

**Figure 12:**
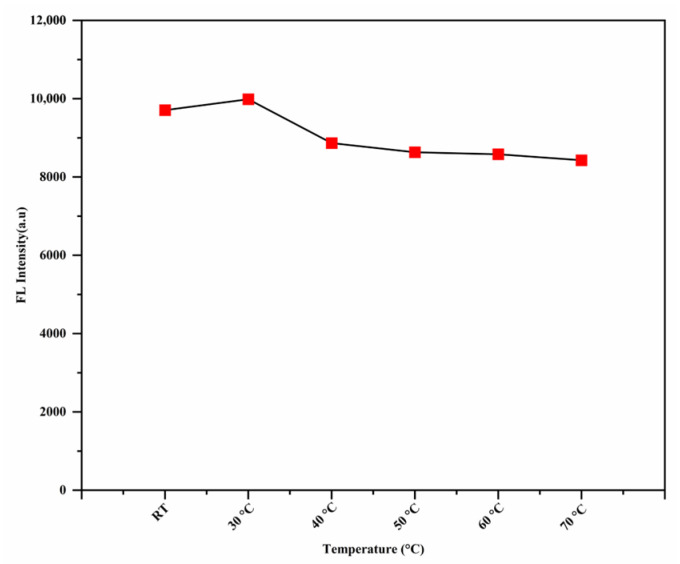
Temperature effects of the RhB-DCT fluorescent probe for Fe^3+^ ions detection.

The effect of temperature on the fluorescence response of the RhB-DCT probe was systematically examined to assess its sensitivity across different thermal conditions. Before measurements, the sample was incubated in a hot water bath for 10 min to achieve thermal equilibrium. As shown in Figure 12, at room temperature or lower, the probe demonstrated increased fluorescence intensity, signifying optimal performance for Fe^3+^ detection. As the temperature increased, a significant reduction in fluorescence intensity was observed, indicating a temperature-dependent quenching effect. This behavior may result from heightened molecular motion and possible disruption of the probe’s interaction with Fe^3+^ ions at elevated temperatures, resulting in diminished fluorescence emission. The RhB-DCT probe exhibited enhanced stability and sensitivity at reduced temperatures, rendering it more suitable for use in cooler environments or scenarios where temperature regulation is possible.

### 3.9. Studies on Reversibility

**Figure 13:**
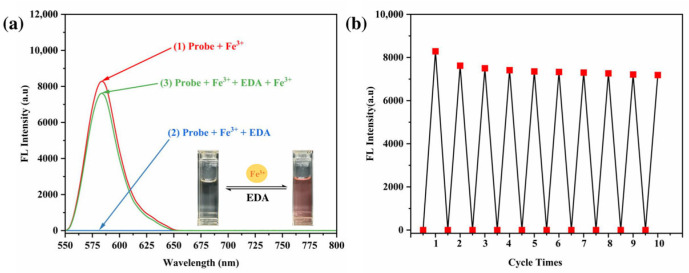
(**a**) Fluorescence reversibility of RhB-DCT probe in an ethanol–water solution (*v*:*v* = 5:5). (**b**) Repeatability cycles of Fe^3+^ detection behavior of RhB-DCT probe.

The reusability and reversibility of the RhB-DCT system for Fe^3+^ recognition was evaluated by introducing ethylenediamine (EDA), a recognized metal-ion chelator, to disrupt the RhB-DCT-Fe^3+^ complex. The incremental addition of EDA resulted in a significant decrease in fluorescence emission intensity, as shown in Figure 13a, indicating the effective sequestration of Fe^3+^ ions by EDA and the disassembly of the complex. The process resulted in the restoration of the original spectral profile of the RhB-DCT probe, demonstrating that the binding interaction was reversible.

The spectral change was accompanied by a visible transformation in solution color, shifting from bright fluorescent pink to nearly colorless, which further confirmed the reformation of the non-fluorescent spirocyclic form of the RhB-DCT molecule. The observations indicate the reversible binding mechanism of RhB-DCT with Fe^3+^ ions, suggesting the probe’s potential for reuse in dynamic sensing applications where reversibility is crucial.

The regenerated RhB-DCT probe retained its responsiveness to Fe^3+^ ions, indicating its suitability for repeated applications. As shown in Figure 13b, the probe’s capacity for reversible detection of Fe^3+^ demonstrates its effectiveness as a recyclable colorimetric sensor. The reusability of this feature markedly improves the probe’s usability in practical applications, especially for the detection of Fe^3+^ ions in aquatic environments. The capacity for regeneration enhances sustainability and provides a cost-effective solution for ongoing environmental monitoring and analytical applications. The ability to regenerate improves sustainability and offers a cost-effective alternative for continuing environmental monitoring and analytical applications.

## 4. Conclusions

In summary, we have developed a highly sensitive and selective Rhodamine B-based “turn-on” fluorescent probe functionalized with 2,4-dichloro-1,3,5-triazine (RhB-DCT) for detecting Fe^3+^ ions in aqueous environments. The RhB-DCT probe exhibited a rapid fluorescence response within seconds following Fe^3+^ binding, along with a notable color transition from colorless to deep pink, facilitating both visual and spectroscopic detection. The probe demonstrated remarkable stability over a broad pH range of 4 to 13 for Fe^3+^ ions detection, was workable at low temperatures, exhibited satisfactory reversibility, and attained a low detection limit of 0.0521 μM. Furthermore, RhB-DCT demonstrated significant selectivity for Fe^3+^ ions, even amidst competing heavy metal ions. The characteristics of the RhB-DCT probe demonstrate its efficacy for real-time monitoring of Fe^3+^ ions in environmental water samples and various complex matrices. This work establishes a significant basis for the advancement of next-generation fluorescent probes aimed at detecting heavy metal ions, thereby enhancing environmental monitoring, public health safety, and analytical sensing technologies.

## Figures and Tables

**Table 1:** Comparison with some other “turn-on” probes for Fe^3+^ ions detection.

Detectable Ions	Ex.λ (nm)	Em.λ (nm)	LOD (μM)	Applications	Ref.
Fe^3+^, Hg^2+^	468	580	0.1	Water Samples	[34]
Fe^3+^	520	582	0.157	Cell Imaging	[35]
Fe^3+^	500	584	0.39	Water Samples	[36]
Fe^3+^	520	580	0.16	Cell Imaging	[37]
Fe^3+^	564	588	2.2	Cell Imaging	[38]
Fe^3+^	520	580	0.083	Water Samples	[39]
Fe^3+^	558	582	0.205	Cell Imaging	[40]
Fe^3+^	520	589	0.27	Water Samples	[41]
Fe^3+^, Cu^2+^	540	589	0.91	-	[42]
Fe^3+^	562	584	0.0521	Water Samples	This work

## Data Availability

The data presented in this study are available upon request.

## Supplementary Materials

The following supporting information can be downloaded at: https://www.mdpi.com/article/10.3390/s25113477/s1, Figure S1. ^1^H NMR spectra of RhB-DCT probe; Figure S2. ^13^C NMR spectra of RhB-DCT probe; Figure S3. FTIR spectra of RhB-DCT probe; Figure S4. Macroscopic pictures under sunlight (up) and ultraviolet light (down) for comparative investigation between Fe^3+^ and other metal ions; Figure S5. The binding constant between RhB-DCT and Fe^3+^ was analyzed using the Benesi–Hildebrand plot; Figure S6. The visual image of RhB-DCT probe under the absence of Fe^3+^ (up) and the presence of Fe^3+^ (down) as a function of pH.
