# Supplementary material for: A Rhodamine B-Based “Turn-On” Fluorescent Probe for Selective Fe3+ Ions Detection
Source: Sensors (Basel). 2025 May 31;25(11):3477. doi: 10.3390/s25113477 (PMC12158345; doi:10.3390/s25113477)
Supplement: Supplementary file 1 [file sensors-25-03477-s001.zip › sensors-3647916-supplementary.pdf]

Supporting Information

A Rhodamine B-Based "Turn-On" Fluorescent Probe for Selective Fe^{3+} Ions Detection

Md Foridul Islam [1,2], Abdulkadir Zakari Abdulkadir [1,2], Smaher M. Elbayomi [3,4,*], Pengfei Zhang [1,2,*]

[1] Guangdong Key Laboratory of Nanomedicine, CAS-HK Joint Laboratory of Biomaterials, CAS Key Laboratory of Biomedical Imaging Science and System, Institute of Biomedicine and Biotechnology, Shenzhen Institutes of Advanced Technology (SIAT), Chinese Academy of Sciences, Shenzhen 518055, China; humaunahammedforid@gmail.com (M.F.I.); abdulkadirzakariabdulkadir@gmail.com (A.Z.A.)
[2] University of Chinese Academy of Sciences, Beijing 101408, China
[3] Polymer Institute, Slovak Academy of Sciences, Dúbravská Cesta 9, 845 41 Bratislava, Slovakia
[4] Department of Chemistry, Faculty of Science, Damietta University, New Damietta 34517, Damietta, Egypt

* Correspondence: smaher-mosad@savba.sk (S.M.E.); pf.zhang@siat.ac.cn (P.Z.)

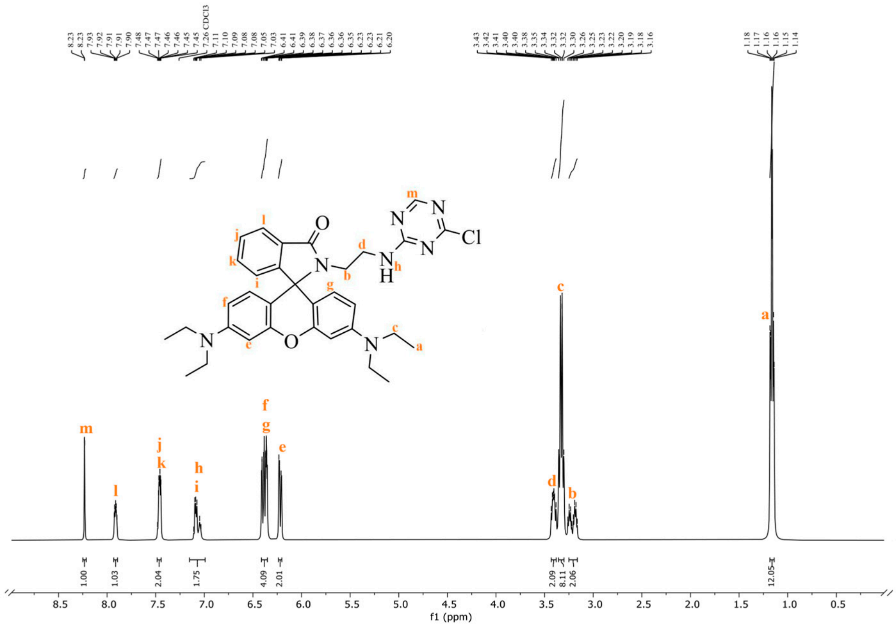

Figure S1. ^1H NMR spectra of RhB-DCT probe.

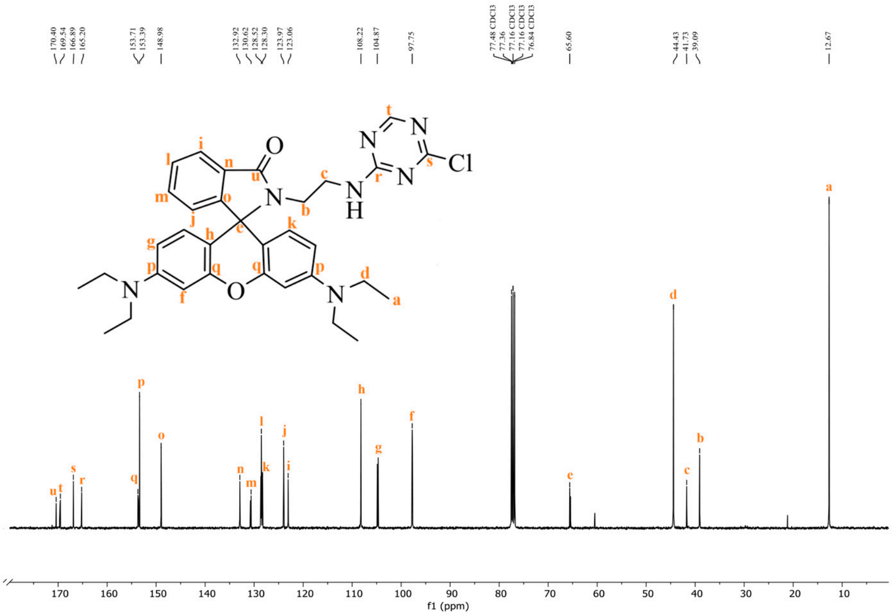

Figure S2. ^{13}C NMR spectra of RhB-DCT probe.

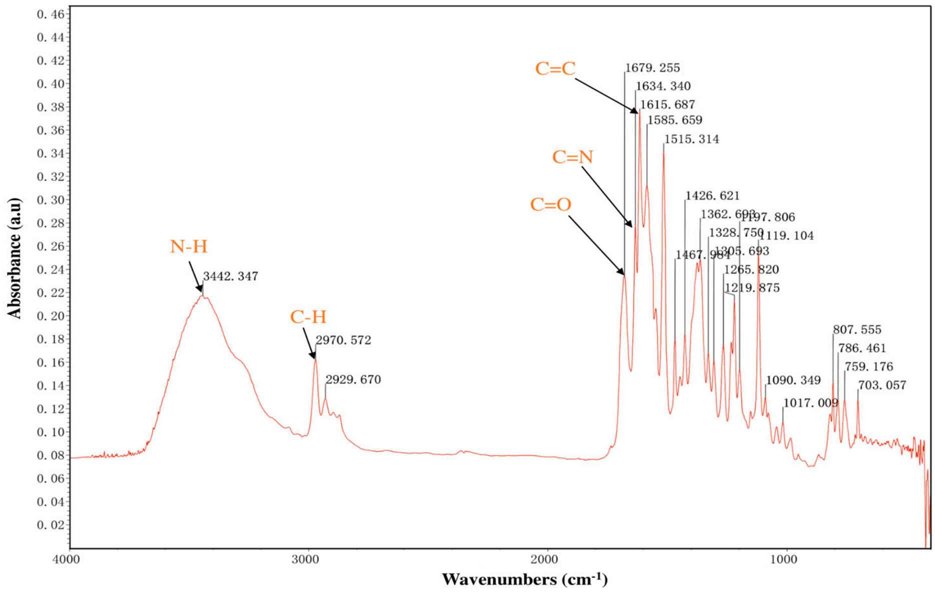

Figure S3. FTIR spectra of RhB-DCT probe.

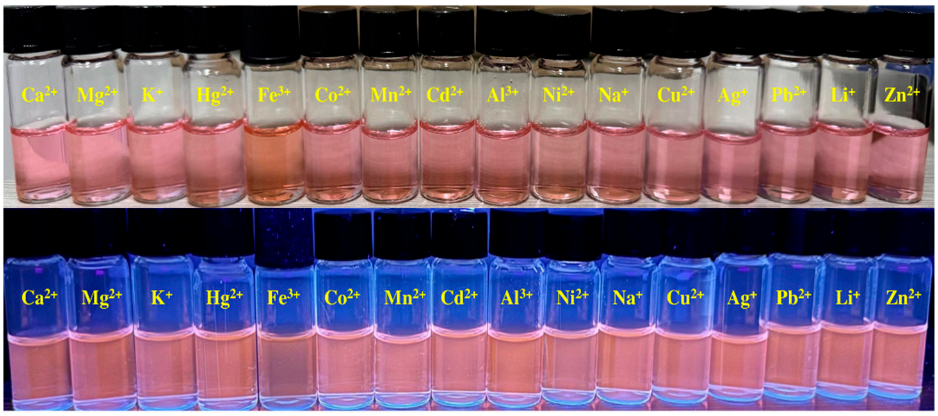

Figure S4. Macroscopic pictures under sunlight (up) and ultraviolet light (down) for comparative investigation between Fe^{3+} and other metal ions.

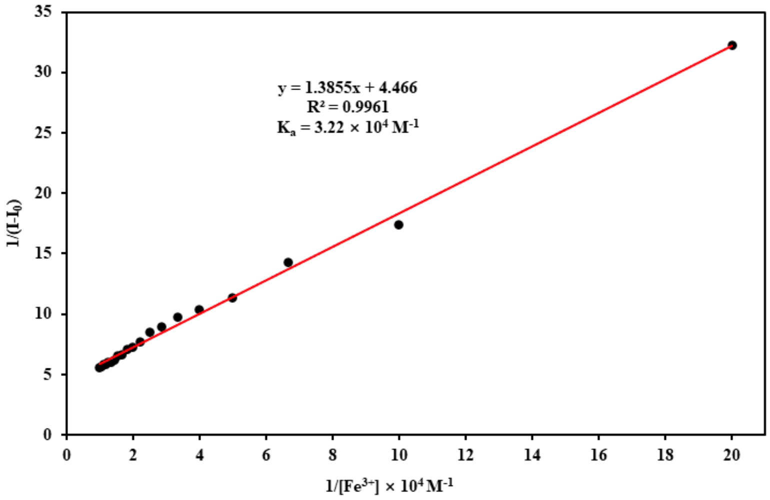

Figure S5. The binding constant between RhB-DCT and Fe^{3+} was analyzed using the Benesi–Hildebrand plot.

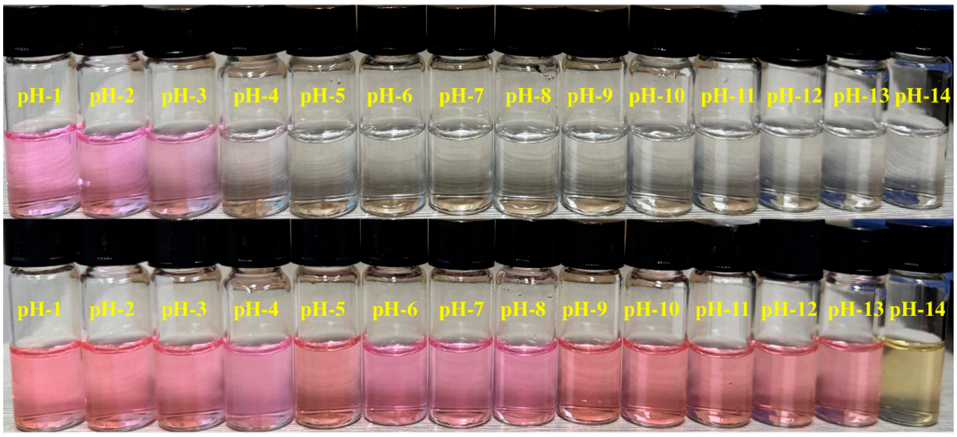

Figure S6. The visual image of RhB-DCT probe under the absence of Fe^{3+} (up) and the presence of Fe^{3+} (down) as a function of pH.
